# Association between dietary inflammatory index and Bell’s palsy: a retrospective case–control study

**DOI:** 10.1007/s00405-026-10305-w

**Published:** 2026-05-21

**Authors:** Burak Celik, Basak Yalciner

**Affiliations:** 1 Department of Otorhinolaryngology, Ankara Yildirim Beyazit University School of Medicine, 06800 Ankara, Turkey; 2 Department of Otorhinolaryngology, Ankara Bilkent City Hospital, Ankara, Turkey

**Keywords:** Bell's palsy, Dietary pattern, Case control studies, Inflammation

## Abstract

**Objectives:**

Bell’s palsy (BP) is an acute facial nerve disorder characterized by rapid-onset unilateral paralysis, in which inflammatory and immune-mediated processes are implicated. The Dietary Inflammatory Index (DII) quantifies the inflammatory potential of the diet and reflects systemic inflammatory burden. However, its association with BP has not been previously investigated.

**Methods:**

This retrospective case–control study included 30 patients with newly diagnosed BP and 30 age- and sex-matched controls without a history of facial paralysis. Dietary intake was assessed using a validated food frequency questionnaire, and DII scores were calculated using standardized methodology. Group differences were analyzed using appropriate statistical tests. Multivariable logistic regression evaluated the association between DII and BP after adjustment for age, sex, and body mass index. Receiver operating characteristic (ROC) analysis assessed discriminatory performance.

**Results:**

Patients with BP had significantly higher DII scores than controls (2.54 ± 1.86 vs. 1.18 ± 1.33; *p* = 0.002). The BP group also exhibited higher intakes carbohydrates, total fat, saturated fat, monounsaturated fat, and sodium (all *p* < 0.01). Each 1-unit increase in DII score was independently associated with BP (adjusted OR = 1.70; 95% CI: 1.18–2.47; *p* = 0.005). ROC analysis demonstrated moderate discriminatory ability (AUC = 0.753; 95% CI: 0.626–0.879).

**Conclusion:**

Higher dietary inflammatory potential is associated with Bell’s palsy, indicating a more pro-inflammatory dietary profile; however, causality cannot be inferred.

## Introduction

Bell’s palsy (BP), named after the Scottish anatomist Sir Charles Bell, is the most common acute mono-neuropathy and arises from involvement of the seventh cranial nerve. It affects individuals across multiple ages and both sexes, with an annual incidence ranging from 11.5 to 53.3 per 100,000 persons across multiple populations [1–3]. It represents the most frequent diagnosis associated with facial nerve weakness or paralysis. BP is an idiopathic, rapidly developing, predominantly unilateral facial nerve paresis (partial weakness) or paralysis (complete loss of movement). This condition leads to partial or complete impairment of voluntary movement of the facial muscles on the affected side. Although typically self-limiting, the resulting facial paresis or paralysis may produce marked temporary oral insufficiency and inability to close the eyelid, thereby predisposing the patient to potential ocular injury.

BP is a multifactorial disorder in which anatomical narrowing, viral reactivation, ischemic injury, immune–inflammatory processes, and cold stimulation act as interrelated mechanisms. The concept of a viral etiology is supported by studies demonstrating that various viruses (HSV-1, HSV-2, VZV, and COVID-19) can remain latent in the geniculate ganglion and, upon reactivation, induce demyelination of the facial nerve [1, 4]. The immune response triggered by viral infection is characterized by infiltration of small round inflammatory cells, degradation of the myelin sheath, and elevated levels of pro-inflammatory cytokines (IL-1, IL-6, TNF-α), resulting in an acute demyelinating process. This inflammatory mechanism leads to Schwann cell injury and rapid conduction block. Systemic inflammatory markers—such as an increased neutrophil-to-lymphocyte ratio—further support the severity of the disease and its immune-mediated neuropathic component [5].

The Dietary Inflammatory Index (DII) is a literature-derived tool designed to quantify the inflammatory potential of an individual’s diet. First proposed in 2014 by Shivappa and colleagues, the DII has been validated as an indicator capable of reflecting systemic inflammatory status. Its scores have been shown to correlate closely with the expression levels of circulating inflammatory biomarkers, including C-reactive protein (CRP), tumor necrosis factor (TNF)-α, interleukin (IL)-1β, IL-6, and IL-10 [6]. At present, the DII is widely applied to examine the contribution of diet-induced inflammation to the onset and progression of various diseases.

Given the central role of inflammation in the pathophysiology of BP and the established capacity of the DII to quantify diet-related inflammatory burden, evaluating the association between DII scores and a history of facial paralysis may provide novel insights into the potential contribution of diet-induced inflammation to this condition. Consequently, the present study aims to investigate the relationship between the inflammatory potential of the diet, as measured by the DII, and the presence or absence of a prior episode of facial paralysis, representing one of the first attempts in the literature to explore this association.

## Methods

### Study design and participants

This study was designed as a retrospective case–control investigation. Participants in the patient group were restricted to individuals with a newly diagnosed (within the preceding three weeks) and clinically confirmed BP, in order to capture the acute or subacute phase of the disease and minimize recall bias in dietary assessment. Diagnosis was verified through medical records and clinician-confirmed evaluation.

Bell’s palsy was diagnosed based on clinical evaluation and confirmed medical records, following standard diagnostic criteria as a diagnosis of exclusion. Patients with alternative causes of facial paralysis (e.g., central neurological disorders, structural lesions, or active infections) were excluded based on clinical assessment and available medical records.

All patients were treated according to the institutional standard protocol, which included oral corticosteroid therapy (1 mg/kg) initiated at diagnosis and tapered over 10–14 days. Antiviral therapy was not administered. In addition, patients were advised to perform facial massage and exercise.

To reduce selection bias and enhance comparability, the control group was selected via simple random sampling from individuals admitted to the Otorhinolaryngology department for elective surgical procedures—such as septoplasty, endoscopic sinus surgery, or tympanoplasty—who had no history of facial paralysis, neurological disease, or systemic inflammatory conditions that could influence dietary patterns or inflammatory status.

Exclusion criteria for both groups included chronic inflammatory or autoimmune disorders, active infection, pregnancy or lactation, and the use of anti-inflammatory medications or corticosteroids within the preceding three months, given their potential to independently alter systemic inflammatory status. To further enhance comparability and reduce potential confounding, frequency matching was performed at the group level based on age, sex, and the presence of common comorbidities, including hypertension, diabetes mellitus, and thyroid disease.

A total of 34 patients with newly diagnosed Bell’s palsy were initially assessed for eligibility. Four patients were excluded due to incomplete or implausible dietary data (*n* = 3) or not meeting the inclusion criteria (*n* = 1). The final Bell’s palsy group consisted of 30 patients.

An age- and comorbidity-matched control group of 30 individuals without a history of facial paralysis was selected.

### Data collection and assessment of facial paralysis history

Sociodemographic characteristics, medical history, and lifestyle-related variables were collected through structured face-to-face interviews conducted by trained researchers. Lifestyle variables included smoking status and physical activity level. A history of BP was confirmed using medical records and clinician-documented diagnoses.

### Dietary assessment and calculation of the DII

Dietary intake was assessed using a validated Food Frequency Questionnaire (FFQ) designed to capture participants’ habitual dietary consumption over the preceding month. Reported intakes were converted into daily consumption frequencies and standardized accordingly. Portion sizes were standardized based on the reference measures implemented within the nutritional analysis software, and daily consumption amounts were calculated for each food item. Subsequently, dietary intake data were converted into nutrient intakes using the Nutrition Information Systems Package Program (BeBiS).

Nutrient and food component data were then processed to calculate individual DII scores in accordance with the standardized methodology developed by Shivappa et al. All dietary parameters available within the DII algorithm were included in the calculation, with higher DII scores indicating a more pro-inflammatory dietary profile.

When available in medical records, clinical data related to metabolic or inflammatory status were extracted to allow consideration of potential confounding variables in the analyses.

### Statistical analysis

All analyses were conducted using appropriate statistical software. Continuous variables were tested for normality and expressed as means ± standard deviations or medians with interquartile ranges, depending on distribution. Categorical variables were presented as frequencies and percentages. Differences in DII scores between individuals with and without a history of facial paralysis were assessed using independent-sample t tests or Mann–Whitney U tests. Logistic regression models were constructed to evaluate the association between DII and the presence of prior facial paralysis, adjusting for age, sex, and body mass index. Statistical significance was defined as *p* < 0.05.

No subgroup or interaction analyses were performed due to the limited sample size. Missing data were handled by complete-case analysis, as participants with incomplete or implausible dietary data were excluded prior to analysis. As this was a matched case–control study, matching was performed at the design stage based on age, sex, and comorbidities, and no additional statistical adjustment for matching was required. No sensitivity analyses were conducted.

An a priori power analysis was conducted based on data from a previously published study [7]. Assuming a two-sided independent samples t-test with an alpha level of 0.05 and an expected effect size derived from the reference study, the power of the present study was calculated using a total sample size of 60 participants (30 per group). Under these assumptions, the achieved statistical power was ≈90% (89.2%), indicating that the current sample size is sufficient to detect a statistically significant difference with high confidence.

## Results

A total of 60 participants were included in the study, comprising 30 individuals with BP and 30 controls without BP. Baseline demographic and anthropometric characteristics are presented in Table 1. There were no statistically significant differences between the BP and non-BP groups in terms of age, sex distribution, height, body weight, body mass index (BMI), or smoking status (all *p* > 0.05). In contrast, the DII score was significantly higher in the BP group compared with the non-BP group (2.54 ± 1.86 vs. 1.18 ± 1.33, *p* = 0.002). According to the House–Brackmann grading system, among the 30 patients with BP, 11 (36.7%) were classified as grade II, 10 (33.3%) as grade III, 6 (20.0%) as grade IV, and 3 (10.0%) as grade V. Reasons for exclusion at each stage of participant selection are presented in Figure S1. There were no missing data for the variables included in Tables 1, 2, 3 and 4 after exclusion of participants with incomplete dietary records.

**Table 1:** Baseline demographic and clinical characteristics of participants with and without Bell’s palsy

	Non-BP (N:30)	BP (N:30)	*p*
Age, years	42.73 ± 13.28	42.47 ± 12.90	0.937
Sex, male, *n* (%)	16 (53.3)	18 (60.0)	0.795†
Height (cm)	172.07 ± 10.55	173.20 ± 10.37	0.676
Weight (kg)	81.57 ± 14.70	81.80 ± 15.89	0.953
BMI (kg/m^2^)	27.49 ± 4.26	27.07 ± 3.51	0.676
Current smoker (yes/no) (*n*)	10/20	13/17	0.596†
DII	1.18 ± 1.33	2.54 ± 1.86	**0.002**

Continuous variables were compared using independent samples t tests; categorical variables were analyzed using Fisher’s exact test (†).*BP* Bell's Palsy; *BMI* Body Mass Index; *DII* Dietary Inflammatory Index

**Table 2:** Comparison of dietary inflammatory index and daily nutrient intakes between participants with and without Bell’s palsy

	Non-BP (N:30)	BP (N:30)	*p*
DII	1.18 ± 1.33	2.54 ± 1.86	**0.002**
Carbohydrates (g/day)	93.52 ± 27.41	138.70 ± 30.33	** < 0.001**
Energy from carbohydrates (%)	40.90 ± 8.56	43.91 ± 7.46	0.08
Protein (g/day)	45.49 ± 25.73	50.87 ± 14.96	0.25
Protein E% (%)	20.02 ± 5.29	16.83 ± 3.10	**0.02**
Fat (g/day)	35.23 ± 15.05	56.77 ± 20.61	** < 0.001**
Fat E% (%)	35.15 ± 6.02	37.98 ± 6.70	0.07
Fiber (g/day)	14.10 ± 6.04	16.60 ± 4.94	0.07
Cholesterol (mg/day) †	138.43 (56.78–311.55)	134.65 (68.58–211.91)	0.61
Saturated fat (g/day)	14.44 ± 6.33	23.68 ± 9.98	** < 0.001**
Monounsaturated fat (MUFA) (g/day)	12.18 ± 4.76	19.12 ± 6.58	**0.003**
Omega-3 fatty acids (g/day) †	0.85 (0.66–0.98)	1.22 (0.95–1.89)	0.06
Vitamin A (mcg/day)	844.75 ± 351.62	1015.27 ± 532.42	0.15
Thiamine (mg/day)	0.58 ± 0.32	0.67 ± 0.14	0.18
Riboflavin (mg/day) †	0.86 (0.56–1.26)	1.14 (0.84–1.54)	0.09
Niacin (mg/day) †	6.83 (3.33–8.96)	7.38 (5.34–10.05)	0.11
Vitamin B12 (mcg/day)	3.34 ± 2.51	3.39 ± 1.69	0.92
Vitamin C (mg/day) †	104.54 (63.97–157.42)	85.37 (56.66–139.16)	0.21
Vitamin D (mcg/day) †	1.67 (0.62–3.23)	1.12 (0.44–2.23)	0.35
Folate (mcg/day)	253.37 ± 86.16	301.03 ± 101.28	0.06
Zinc (mg/day) †	4.81 (3.17–12.57)	7.00 (4.90–9.74)	0.09
Calcium (mg/day) †	556.64 (300.42–839.17)	765.52 (486.15–962.69)	0.07
Sodium (mg/day)	1835.25 ± 527.92	2732.48 ± 891.19	**0.002**
Magnesium (mg/day)	178.65 ± 144.96	208.50 ± 75.77	0.14
Iron (mg/day) †	5.06 (3.28–6.81)	6.88 (4.93–8.27)	0.08
† Median (IQR); Mann–Whitney U test

**Table 3:** Distribution of comorbid conditions among participants with and without Bell’s palsy

	Non-BP (N:30)	BP (N:30)	*p*
Hypertension, *n* (%)	8(%26)	9(%30)	0.779
Diabetes mellitus, *n* (%)	4(%13)	5(%16)	0.723
Coronary artery disease *n* (%)	2(%6)	2(%6)	1
COPD- Asthma *n* (%)	3(%10)	2(%6)	0.398
Thyroid Disease	1(%3)	2(%6)	0.561

*COPD* Chronic obstructive pulmonary disease

**Table 4:** Comparison of DII according to Bell’s palsy severity

Variable	Mild (HB II–III) (*n* = 21)	Severe (HB IV–V) (*n* = 9)	Z	*p*
DII	1.5 (0.9–2.4)	2.6 (1.2–3.8)	-1.97	0.049

Values are presented as median (interquartile range). Comparisons were performed using the Mann–Whitney U test

Daily nutrient intakes according to BP status are shown in Table 2. Carbohydrate intake was also significantly higher in the BP group (138.70 ± 30.33 g/day vs. 93.52 ± 27.41 g/day, *p* < 0.001). Total fat intake (56.77 ± 20.61 g/day vs. 35.23 ± 15.05 g/day, *p* < 0.001), saturated fat intake (23.68 ± 9.98 g/day vs. 14.44 ± 6.33 g/day, *p* < 0.001), monounsaturated fatty acid (MUFA) intake (19.12 ± 6.58 g/day vs. 12.18 ± 4.76 g/day, *p* = 0.003), and sodium intake (2732.48 ± 891.19 mg/day vs. 1835.25 ± 527.92 mg/day, *p* = 0.002) were all significantly higher in the BP group. Protein intake did not differ significantly between groups (50.87 ± 14.96 g/day vs. 45.49 ± 25.73 g/day, *p* = 0.25); however, the percentage of energy derived from protein was significantly lower in the BP group (16.83 ± 3.10% vs. 20.02 ± 5.29%, *p* = 0.02). No statistically significant differences were observed between groups for energy derived from carbohydrates, fat-derived energy percentage, fiber intake, omega-3 fatty acid intake, cholesterol intake, or micronutrient intakes including vitamins A, B1, B2, B3, B12, C, and D, as well as calcium, zinc, magnesium, folate, and iron (all *p* > 0.05).

The distribution of comorbid conditions among participants is presented in Table 3. There were no statistically significant differences between the BP and non-BP groups with respect to the prevalence of hypertension, diabetes mellitus, coronary artery disease, chronic obstructive pulmonary disease/asthma, or thyroid disease (all *p* > 0.05).

DII scores were higher in patients with severe Bell’s palsy compared to those with mild disease (*p* = 0.049); however, this subgroup analysis should be considered exploratory due to the relatively small sample size (Table 4).

Binary logistic regression analysis examining factors associated with BP is presented in Table 5. After adjustment for age, sex, and BMI, DII remained independently associated with BP. Each 1-unit increase in DII score was associated with higher odds of BP (adjusted odds ratio [OR]: 1.70; 95% confidence interval [CI]: 1.18–2.47; *p* = 0.005). Age, sex, and BMI were not significantly associated with BP in the adjusted model (all *p* > 0.05).

**Table 5:** Binary logistic regression analysis of factors associated with BP

	Adjusted OR (95% CI)	*p*
DII (per 1-unit)	1.70 (1.18–2.47)	**0.005**
Age, years	1.00 (0.96–1.05)	0.861
Sex, male	0.75 (0.24–2.31)	0.611
BMI (kg/m^2^)	0.98 (0.84–1.13)	0.747

*BP* Bell's Palsy; *BMI* Body Mass Index; *DII* Dietary Inflammatory Index

**Fig. 1:**
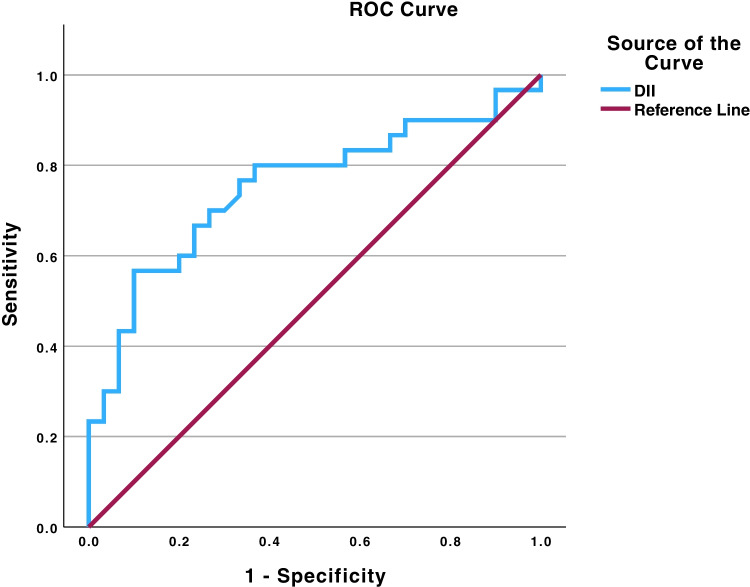
Receiver operating characteristic (ROC) curve of the Dietary Inflammatory Index (DII) for discriminating Bell’s palsy cases from controls

Receiver operating characteristic (ROC) curve analysis was performed to assess the discriminatory performance of DII for classifying BP status (Fig. 1). The area under the ROC curve (AUC) was 0.753 (standard error: 0.065; 95% CI: 0.626–0.879; *p* < 0.001). Using Youden’s index, a DII cutoff value of ≥ 1.48 yielded a sensitivity of 80.0% and a specificity of 63.3% for classifying BP.

## Discussion

To our knowledge, this study is among the first to examine the association between diet-related inflammation and BP. We observed that individuals with a history of BP had significantly higher DII scores—reflecting a more pro-inflammatory dietary pattern—compared with individuals without a history of facial paralysis. Previous research on BP has predominantly focused on viral, anatomical, and immune-mediated mechanisms, while the potential role of dietary factors has received little attention. By demonstrating an association between a pro-inflammatory dietary profile and BP, the present findings address an important gap in the existing literature and suggest that modifiable dietary factors may be associated with the inflammatory milieu observed in BP; however, causal relationships cannot be established.

Dietary factors are major modifiable determinants of chronic low-grade inflammation, and the DII was developed to quantify this inflammatory potential. Higher DII scores have been shown to correlate with circulating inflammatory mediators such as C-reactive protein, interleukin-6, and tumor necrosis factor-α [8]. Therefore, our findings should be interpreted as reflecting the *potential inflammatory burden of diet* rather than direct biological evidence of systemic inflammation. Pro-inflammatory dietary patterns have been shown to promote systemic inflammation through increased oxidative stress, immune dysregulation, and enhanced production of inflammatory cytokines. Diets rich in saturated fats and refined carbohydrates, characteristic of Western dietary patterns, are consistently associated with higher levels of inflammatory markers, whereas healthier dietary patterns such as the Mediterranean diet—rich in fruits, vegetables, and fiber—are linked to lower inflammatory burden [9]. The DII was specifically developed to capture these cumulative effects of diet on inflammation and has been widely applied in diverse clinical settings. Consistent associations between higher DII scores and increased risk of inflammation-related conditions, including metabolic [10], oncological [11], and neuropsychiatric disorders [12], have been reported across multiple populations. Within this context, the higher DII scores and more pro-inflammatory dietary profile observed in our BP cohort further support the relevance of diet-induced inflammation as a potential contributor to inflammatory disease processes.

Our findings are consistent with a growing body of evidence linking pro-inflammatory dietary patterns to adverse outcomes across a broad spectrum of inflammation-related conditions. Elevated DII scores have been associated with increased disease prevalence or activity in chronic inflammatory disorders such as endometriosis [13] and rheumatoid arthritis [14], as well as with higher levels of systemic inflammatory markers. Similar associations have also been reported in autoimmune [15] and neuroinflammatory [16] conditions, supporting the role of diet-related inflammation in modulating immune-mediated disease processes. Importantly, emerging evidence suggests that the inflammatory potential of the diet may also influence outcomes in acute inflammatory conditions. In this context, recent evidence from a systematic review and dose–response meta-analysis has demonstrated that higher DII scores are independently associated with an increased risk of SARS-CoV-2 infection and greater COVID-19 severity, while evidence regarding mortality remains limited, highlighting the potential role of baseline dietary inflammatory burden in shaping host inflammatory responses during acute infectious diseases [17]. Collectively, these findings suggest that diet-induced inflammation may contribute to an unfavorable systemic inflammatory milieu in both chronic and acute settings. Within this broader framework, the observed association between a pro-inflammatory dietary profile and BP—an acute inflammatory neuropathy—appears biologically plausible and aligns with evidence from inflammation-driven conditions. Consistent with this broader body of evidence, our study similarly demonstrated that individuals with BP exhibited significantly higher DII scores compared with controls, indicating a more pro-inflammatory dietary profile among patients with a history of facial paralysis.

Elevated neutrophil-to-lymphocyte ratio (NLR) have been shown to correlate with poor outcomes in a variety of clinical settings, including malignancy, sepsis,and severe infections [18–21]. Peripheral inflammatory markers, particularly the NLR, have been consistently reported to be elevated in patients with BP and to be associated with poorer clinical outcomes, indicating a link between systemic neutrophil-predominant inflammation and neural injury [22]. Supporting this association, higher NLR values have also been observed in patients demonstrating contrast enhancement of the facial nerve on magnetic resonance imaging, a radiological finding indicative of active nerve inflammation [23]. These observations further suggest that elevated systemic inflammatory burden may be reflected in both peripheral blood markers and local neural inflammatory changes. Taken together, these findings suggest that the degree of systemic inflammation may influence the radiological detectability of facial nerve inflammation. In states of heightened inflammatory burden, reflected by elevated NLR values, neuritis of the facial nerve may be more pronounced and therefore more readily detectable as contrast enhancement on magnetic resonance imaging. Conversely, in individuals with a lower systemic inflammatory profile, inflammatory changes within the facial nerve may be subtler and fall below the threshold of radiological detection, despite the clinical presence of BP. This may partly explain why facial nerve enhancement is not consistently observed in all patients with BP and supports the notion that radiological findings may reflect the intensity of the underlying inflammatory process rather than its mere presence. When patients were stratified according to disease severity, DII scores were higher in patients with severe Bell’s palsy compared to those with mild disease (*p* = 0.049). This finding is consistent with previous studies demonstrating a positive association between disease severity and inflammatory markers, such as the NLR. Accordingly, the higher DII scores observed in our BP cohort support a potential link between pro-inflammatory dietary patterns and systemic inflammatory burden. However, this analysis should be interpreted with caution, as it is exploratory in nature and limited by the relatively small number of patients in each subgroup.

Nutritional status has also been linked to disease severity in BP. Vitamin D, a nutrient with well-established immunomodulatory and neuroprotective properties, has been shown to be significantly lower in patients with severe BP compared with healthy controls, whereas no significant differences have been observed in patients with milder disease. In line with these findings, a recent systematic review and meta-analysis demonstrated that reduced serum vitamin D levels were specifically associated with higher disease grades, while evidence regarding disease incidence remained inconclusive [24]. Together, these observations suggest that insufficient vitamin D status may exacerbate neural inflammation and injury rather than directly influence the onset of BP. Collectively, this evidence supports the biological plausibility of our findings by indicating that a pro-inflammatory dietary pattern—characterized by lower intake of anti-inflammatory nutrients such as vitamin D—may contribute to an unfavorable systemic inflammatory milieu, whereas an anti-inflammatory diet may help mitigate inflammatory burden affecting the facial nerve. In our study, dietary vitamin D intake did not differ significantly between individuals with BP and controls, suggesting that the observed association between DII and BP is unlikely to be driven by vitamin D intake alone and may instead reflect the cumulative inflammatory potential of the overall dietary pattern. This lack of difference may partly be explained by the clinical severity distribution of our cohort, as approximately 70% of patients presented with mild-to-moderate disease (House–Brackmann grades II–III), a stage at which systemic inflammatory burden may be relatively limited.

### Limitations

Several limitations of the present study should be acknowledged. First, the case–control design precludes causal inference; therefore, the observed association between dietary inflammatory potential and Bell’s palsy cannot establish a temporal or causal relationship. Prospective longitudinal studies are warranted to clarify whether pro-inflammatory dietary patterns contribute to disease onset or progression.

Second, dietary intake was assessed using a food frequency questionnaire, which is subject to recall bias and measurement error, despite efforts to minimize this limitation by restricting inclusion to newly diagnosed patients and employing a validated assessment tool.

Third, systemic inflammatory biomarkers—such as C-reactive protein, interleukins, or neutrophil-to-lymphocyte ratio—were not directly measured. As a result, the inflammatory burden was inferred indirectly through the Dietary Inflammatory Index rather than confirmed by laboratory parameters. Future studies integrating dietary indices with circulating inflammatory biomarkers would provide more comprehensive insight into the underlying mechanisms.

In addition, the relatively modest sample size and single-center design may limit the generalizability of the findings. Additionally, the subgroup analysis according to disease severity should be considered exploratory due to the limited number of patients in each subgroup. The control group consisted of patients undergoing elective otorhinolaryngological procedures (e.g., septoplasty), who were considered to broadly reflect individuals without systemic inflammatory conditions; however, they may not fully reflect the general population. To improve comparability, controls were frequency-matched to cases based on comorbid conditions. However, as the controls were hospital-based, this approach may introduce potential selection bias. In addition, given the number of statistical comparisons performed, the possibility of type I error cannot be excluded, and therefore the findings should be interpreted with appropriate caution.

Furthermore, several lifestyle-related variables (e.g., physical activity and socioeconomic status) were not included in the regression model due to the limited sample size, and residual confounding cannot be excluded.

Finally, although matching was applied, residual confounding by unmeasured factors cannot be fully excluded.

## Conclusion

In conclusion, this retrospective case–control study demonstrates that individuals with Bell’s palsy exhibit significantly higher Dietary Inflammatory Index scores compared with controls, indicating a more pro-inflammatory dietary pattern. These findings support a potential association between diet-related inflammatory burden and Bell’s palsy and provide preliminary evidence that dietary factors may be relevant to the inflammatory milieu underlying this acute neuropathy. Although causality cannot be inferred, the results highlight the importance of considering overall dietary inflammatory potential in the context of Bell’s palsy and underscore the need for prospective studies integrating dietary assessment with inflammatory biomarkers to further elucidate this relationship.

## Data Availability

The data that support the findings of this study are available from the corresponding author upon reasonable request.

## Declarations

### Statement on the use of generative artificial intelligence and AI-assisted technologies in the writing process

Artificial intelligence tools were used solely for language editing and enhancement during the writing process of this manuscript; all data analysis, result interpretation, and scientific content development were conducted by the authors.
